# Supplementary figures and images for: Photosynthetic sea slugs induce protective changes to the light reactions of the chloroplasts they steal from algae
Source: eLife. 2020 Oct 20;9:e57389. doi: 10.7554/eLife.57389 (PMC7679141; doi:10.7554/eLife.57389)

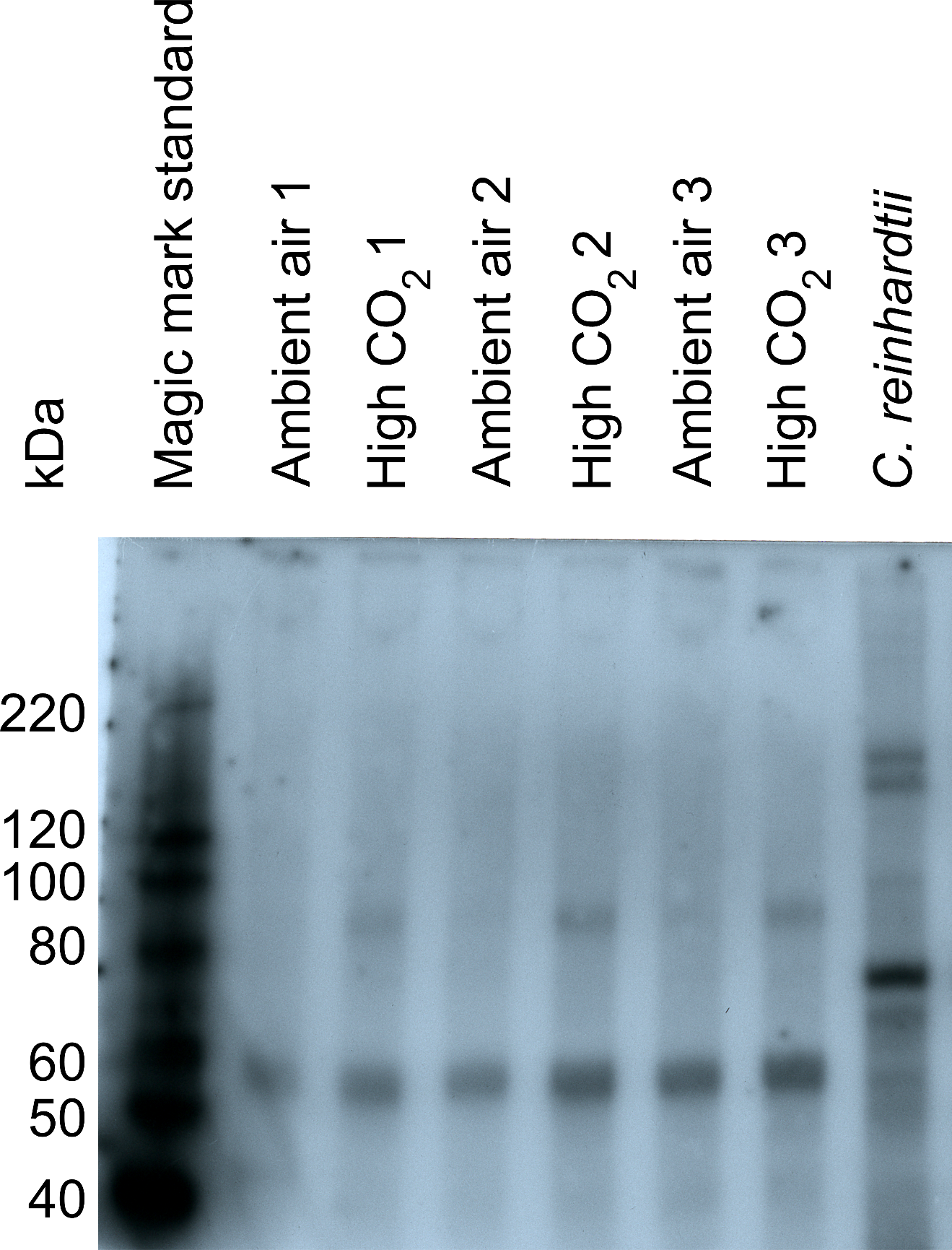

Supplement: Figure 6—source data 1. [file elife-57389-fig6-data1.docx]
